# Mutations on a conserved distal enhancer in the porcine C-reactive protein gene impair its expression in liver

**DOI:** 10.3389/fimmu.2023.1250942

**Published:** 2023-09-14

**Authors:** Carles Hernández-Banqué, Teodor Jové-Juncà, Daniel Crespo-Piazuelo, Olga González-Rodríguez, Yuliaxis Ramayo-Caldas, Anna Esteve-Codina, Marie-José Mercat, Marco C. A. M. Bink, Raquel Quintanilla, Maria Ballester

**Affiliations:** ^1^ Animal Breeding and Genetics Program, Institute of Agrifood Research and Technology (IRTA), Caldes de Montbui, Spain; ^2^ CNAG-CRG, Centre for Genomic Regulation (CRG), Barcelona Institute of Science and Technology (BIST), Barcelona, Spain; ^3^ Universitat Pompeu Fabra (UPF), Barcelona, Spain; ^4^ IFIP-Institut Du Porc and Alliance R&D, Le Rheu, France; ^5^ Hendrix Genetics Research, Technology & Services B.V., Boxmeer, Netherlands

**Keywords:** C-reactive protein, fine-mapping, luciferase assays, enhancer, pig, RNA-seq

## Abstract

C-reactive protein (CRP) is an evolutionary highly conserved protein. Like humans, CRP acts as a major acute phase protein in pigs. While *CRP* regulatory mechanisms have been extensively studied in humans, little is known about the molecular mechanisms that control pig *CRP* gene expression. The main goal of the present work was to study the regulatory mechanisms and identify functional genetic variants regulating *CRP* gene expression and CRP blood levels in pigs. The characterization of the porcine *CRP* proximal promoter region revealed a high level of conservation with both cow and human promoters, sharing binding sites for transcription factors required for *CRP* expression. Through genome-wide association studies and fine mapping, the most associated variants with both mRNA and protein CRP levels were localized in a genomic region 39.3 kb upstream of *CRP*. Further study of the region revealed a highly conserved putative enhancer that contains binding sites for several transcriptional regulators such as STAT3, NF-kB or C/EBP-β. Luciferase reporter assays showed the necessity of this enhancer-promoter interaction for the acute phase induction of *CRP* expression in liver, where differences in the enhancer sequences significantly modified *CRP* activity. The associated polymorphisms disrupted the putative binding sites for HNF4α and FOXA2 transcription factors. The high correlation between *HNF4α* and *CRP* expression levels suggest the participation of HNF4α in the regulatory mechanism of porcine *CRP* expression through the modification of its binding site in liver. Our findings determine, for the first time, the relevance of a distal regulatory element essential for the acute phase induction of porcine *CRP* in liver and identify functional polymorphisms that can be included in pig breeding programs to improve immunocompetence.

## 1 Introduction

The C-reactive Protein (CRP) is an evolutionary well-conserved protein that plays a significant role in the acute phase response to inflammation. This protein belongs to the pentraxins family and has two conformations: native CRP (nCRP) and monomeric CRP (mCRP). Although this protein is mainly produced by hepatocytes, it is also synthesized, in marginal concentrations, in neurons, monocytes, lymphocytes and adipocytes (1).

Depending on the conformation present in any given stage of the inflammatory process, CRP serves as a pro-inflammatory molecule through the activation of the initial stages of the complement system and the modulation of nitric oxide release and cytokine synthesis. Moreover, CRP functions as an anti-inflammatory compound by controlling the progression and intensity of the late stages of inflammation and modulating apoptosis and phagocytosis processes (2–5). CRP protein levels are currently being used as a stable marker in humans for the prediction, prevention and prognosis of cardiovascular disease as well as several other chronic diseases (6–8).

There is sufficient evidence that CRP blood levels are a complex phenotype with several environmental and genetic determinants. External factors such as the weight or the overall stress levels of individuals may potentially modulate the levels of this protein (9, 10). At genetic level, several studies in humans have determined the impact of polymorphisms in *CRP* and in other inflammatory-related genes on CRP blood levels (11–13). In pigs, a genome wide association study (GWAS) in a commercial Duroc population identified the genomic region in which *CRP* is annotated as associated with the variation in its translated protein levels (14). Furthermore, according to several studies, a variety of transcription factors affect the expression of this gene, highlighting the need to further study the interaction and effects of *CRP* regulatory pathways (15–19). While HNF-1, HNF-3, and OCT-1 transcriptions factors are involved in the regulation of basal *CRP* expression levels (20–22), the induction of the acute phase expression of *CRP* is mediated by the effect of cytokines, particularly IL-6, IL-1β and TNF-α, through the activation of STAT3, NF-kB and C/EBP-β transcription factors (16, 19, 23–25). In this regard, a recent study in human Hep3B cells identified an enhancer upstream of the *CRP* promoter enriched in binding sites for STAT3 and C/EBP-β with a major impact on the acute phase induction of *CRP* expression (26).

Considering the high resemblance between pig and human immune responses, understanding the molecular mechanisms that control porcine *CRP* gene expression may further support the use of the pig as a biomedical animal model for the study of human diseases. In other vein, genetic selection for immunity traits in livestock has been proposed as a promising approach for improving animal robustness and disease resistance, thus contributing to healthier livestock while reducing the emergence of antibiotic resistances (27–30).

The present work aimed to study the regulatory mechanisms affecting the expression of porcine *CRP* and identify causal genetic variants that influence CRP levels in blood to better understand the genomic physiology of immunocompetence in pigs.

## 2 Methods

### 2.1 Ethics

All experimental protocols and procedures with pigs were approved by the Institut de Recerca i Tecnologia Agroalimentàries (IRTA) Ethical Committee in accordance with the Spanish Policy for Animal Protection RD53/2013, which meets the European Union Directive 2010/63/EU for the correct practices and protection of the animals used in experimentation.

### 2.2 Animal material and phenotypic parameters

The study was performed with a population of 432 healthy piglets (217 males and 215 females of around 60 days of age) belonging to a commercial Duroc pig line. The pigs came from six batches (72 ± 1 animals per batch) and were raised in the same farm with an *ad libitum* cereal-base commercial diet. At the moment of sampling, the animals did not present any sign of infection or pathology, and no antibiotics were supplied.

Blood was collected via the external jugular vein into vacutainer tubes with or without anticoagulants (Sangüesa S.A., Spain), which required the restraint of the animals but not their sedation. Serum samples, in duplicate, diluted 1:3000 were used to measure CRP levels by ELISA kit (Abcam Plc., Spain) following manufacturer’s instructions. Absorbance was read at 450 nm using an ELISA plate reader (Bio-Rad) and analysed using the Microplate manager 5.2.1 sofware (Bio-Rad). Genomic DNA was extracted from blood samples using the NucleoSpin Blood (Macherey–Nagel, Germany). DNA concentration and purity were measured in a Nanodrop ND-1000 spectrophotometer.

### 2.3 SNP genotyping

The 432 animals of the Duroc population were genotyped with the GGP Porcine HD Array (Illumina, San Diego, CA) using the Infinium HD Assay Ultra protocol. The software PLINK/v1.90b3.42 (31) was used to remove those single-nucleotide polymorphisms (SNPs) with a minor allele frequency (MAF) lower than 5%, SNPs with more than 10% missing genotypes, and SNPs that did not map to the porcine reference genome (Sscrofa11.1). A subset of 42,641 SNPs remained for further analysis. Additionally, the rs327446000 SNP within the *CRP* gene was genotyped for the 432 animals by custom designed Taqman assays in a QuantStudioTM 12K Flex Real-Time PCR System (ThermoFisher Scientific).

### 2.4 Whole genome and RNA sequencing data

Whole-genome sequences from 300 pigs (n=100 Landrace, n=100 Large White, and n=100 Duroc) (32) were used to identify and estimate the segregation of CRP polymorphisms. All DNA samples were sequenced (NovaSeq6000 platform) to a minimum read depth of 10X. DNA sequences were mapped against the reference genome (*Sscrofa11.1* assembly) with BWA-MEM/0.7.17 (33) and 44,127,400 polymorphisms (SNPs and indels) were extracted using the GATK/4.1.8.0 Haplotype Caller (34). After filtering genetic variants with PLINK/v1.90b3.42 software a total of 25,315,878 polymorphisms remained for downstream analysis. Furthermore, the expression levels of *CRP* isoforms in the liver were quantified in RNA-seq data from the same 300 pigs (32). RNA-seq reads were mapped against the reference genome (*Sscrofa11.1* assembly) with STAR/v2.5.3a (35) using ENCODE parameters. Annotated genes and isoforms in Ensembl Genes 101 database were quantified with RSEM/1.3.0 (36) using default parameters.

### 2.5 Identification of polymorphisms in the CRP gene and comparative promoter analysis

SNPs and indels within and in the vicinity of the *CRP* (between positions 90.7-90.8 Mb on SSC4) were extracted from whole genome sequencing (WGS) data. VEP software (37) was used to locate and predict the consequences of variants on the CRP protein sequence using the Ensembl Genes 106 annotation database and the *Scrofa11.1* assembly. The promoter regions of the porcine *CRP* isoforms were aligned to the reference human and cow orthologue promoter sequences annotated in the Ensembl database with the Multalin software (38) in order to measure the level of conservation between them.

A computer-assisted identification of putative transcription factors binding sites in *CRP* regulatory regions (enhancer and proximal promoter) was performed with LASAGNA-Search 2.0 (39).

### 2.6 Polymorphism association analysis

The association between polymorphisms identified in both the regulatory and coding regions of *CRP* and CRP blood levels (n=100 Duroc individuals) was analyzed by using the *aov*() function in R. Normality of CRP data was checked through Shapiro-Wilk test in R, and logarithm transformation was applied to reach normal distribution of residuals (*P*-value > 0.05). Systematic non-genetic putative effects (sex and batch) were tested by using the *lm*() function in R. When significant, sex and/or batch effects were considered for subsequent analyses. Multiple testing corrections were performed with the false discovery rate (FDR) method using the *p.adjust* function in R (40). Significance threshold for the association was set at FDR ≤ 0.05.

### 2.7 Genome wide association study

GWAS was performed using 42,641 SNPs, together with the rs327446000 *CRP* SNP, and the CRP levels in serum of 432 Duroc animals using the GCTA 1.94.1 software (41) with the following model:


$$\left(1\right)\;\;y_{ij}\;=\;b_{j}\;+\;u_{i}\;+\;s_{li}a_{l}\;+\;e_{ij}$$

Where *y_ij_* corresponds to the phenotype (log transformed CRP levels in serum) of the *i* ^th^ individual in the *j* ^th^ batch; *bj* corresponds to the *j* ^th^ batch effect (6 levels); *u_i_* is the infinitesimal genetic effect of individual *i*, with u∼*N*(0, G^2^_*u*_), where G is the genomic relationship matrix (GRM) calculated using the filtered autosomal SNPs based on the methodology of (42) and σ^2^_u_ is the additive genetic variance; *s_li_* is the genotype (coded as 0, 1, 2) for the *l* ^th^ SNP, being *a_l_* its allele substitution effect on the trait under study; and *e_ij_* is the residual term.

The false discovery rate (FDR) method of multiple testing described by Benjamini and Hochberg (40) was used to measure the statistical significance for association studies at genome-wide level with the *p.adjust* function of R. The significant association threshold was set at FDR ≤ 0.05. A Manhattan plot based on the resulting significance was generated using the R package qqman (43).

### 2.8 Chromosome 4 association and fine mapping

WGS data from 100 individuals of the same Duroc population was used to impute genotypes at the whole genome level of the 354 individuals (332 piglets and 22 boars) that had been previously genotyped with the GGP Porcine HD Array.

Imputation and haplotype reconstruction was performed with 19,610 SNPs (MAF ≥ 5%), covering the SSC4:88,251,177-92,759,955 bp genomic region, using DualPHASE/v.2.3 software (44). This haplotype-based approach exploits population (linkage disequilibrium; LD) and family information (Mendelian segregation and linkage analysis; LA) through a Hidden Markov model. Linkage was estimated based on the equivalence 1Mb~1cM.

GWAS for the CRP levels in serum and the 19,610 SNPs was performed using the fastGWA option of the GCTA 1.94.1 (41) software, following the previous model (1). QTL fine-mapping was performed with Qxpak/v5 (45) based on the reconstructed haplotypes to simultaneously exploiting LA and LD with the following mixed model:


$$\left(2\right)\;Y\;=\;X\beta \;+\;Z_{h}h\;+\;Z_{u}u\;+\;e$$

Where β corresponds to the vector containing the batch fixed effect, h is the vector of random QTL effects corresponding to the K cluster defined by the Hidden State (HS), u is the vector of random individual polygenic effects and e is the vector containing the residuals.

Multiple test correction was performed using the Bonferroni method (46). The significant association threshold was set at p adjust ≤ 0.05.

### 2.9 Expression GWAS

Expression GWAS (eGWAS) were performed with a total of 25,315,878 genetic variants and the RNA-seq expression data of each *CRP* isoform in the 300 pigs (n=100 Landrace, n=100 Large White and n=100 Duroc). The association was estimated by fitting the previously described model (1) using the GCTA software (41):


$$\left(3\right)\;\;y_{ijk}\;=\;sex_{j}\;+\;breed_{k}\;+\;u_{i}\;+\;s_{li}a_{l}\;+\;e_{ijk}$$

Where *y_ijk_* corresponds to the gene expression in the *i* ^th^ individual of sex *j* and belonging to the *k^th^* breed (3 levels)*; u_i_, s_li_, a_l_* and *e_ijk_* are as defined in the previous GWAS model. After multiple testing adjustment, association threshold was established at FDR ≤ 0.05. Manhattan plots were generated in R as previously mentioned.

### 2.10 Luciferase assay

Two individuals of the Duroc population with different haplotypes (Haplotype P1: G – T – C – G – C – T – G – A – C and Haplotype P2: A – A – C – A – T – C – A – G – T) for *CRP* proximal promoter polymorphisms were selected for the cloning process. Genomic DNA was used to amplify two fragments of ~600bp corresponding to *CRP* promoter regions of the selected animals, using the forward primer 5’-GAGGATATCAAGATCGATCAAGCACATGTTTCACTGC-3’ and the reverse primer 5’-CCGGATTGCCAAGCTCCCCTTGGAGAAGATGCC-3’, containing the *Hind*III and *Bgl*II restriction sites. Amplification of the fragments was performed by PCR with Hot-Star Taq Master Mix Kit (Qiagen, Spain) under the following conditions: 15 min at 94°C, 35 cycles of 1 min at 94°C, 1 min at 60°C and 1 min at 72°C and a final extension of 10 min at 72°C.

PCR products were cloned into pNL1.2[NlucP] vector (Promega, Spain) with the In-Fusion Snap Assembly cloning kit (Takara, Japan). Plasmids were purified using PureYield™ Plasmid Miniprep System kit (Promega, Spain). Enhancer region fragments of ~280bp for the same individuals (Haplotype E1: G – C – T – Ø – A – A – G – C – G and Haplotype E2: A – A – C – TTCTGTTTGTGGGACCGGCCC – G – G – A – T – T) were amplified by PCR using the forward primer 5’-GCTCGCTAGCCTCGAGTGGAAGAGAGGGTGGGGTG-3’ and the reverse primer 5’-TTGATCGATCTTGATATCGCAGCTACCTCAGAACACAGTC-3’, containing the XhoI and EcoRV restriction sites, with Hot-Star Taq Master Mix Kit (Qiagen, Spain) and the previous conditions, and were inserted upstream of the promoter regions of the corresponding haplotypes. Nucleotide sequence of each DNA construct was confirmed by Sanger sequencing.

HepG2 cells (ATCC, USA) were seeded at a density of 3x10^4^ cells per well in a 96 wells plate in DMEM supplemented with 10% inactivated fetal bovine serum, 1% penicillin-streptomycin, 1% L-glutamine and 1% pyruvate. After 24h cells were cotransfected with 500ng of pNL1.2[NlucP] vector, 12.5ng of pGL4.54[luc2/TK] and 487.5ng of transfection carrier DNA (Promega, Spain) using ViaFect™ Transfection Reagent (3:1) (Promega, Spain) in serum-free medium for 16h. Cells were then treated with IL-6 (10ng/ml) and IL-1β (1ng/ml). Luciferase activity measurements were performed 24h after stimulation with Dual-Luciferase Reporter Assay System (Promega, Spain). Expression of nanoluc luciferase driven by inserted promoters and enhancers was normalized to the cotransfected firefly control vectors. Every luciferase assay was made by triplicate in different days with three replicates for each vector in each experiment to increase the robustness of the results.

## 3 Results

### 3.1 Comparative study of *CRP* proximal promoter region between human, pig and cow and identification of polymorphisms in the porcine *CRP* gene

Since human *CRP* gene has been proved to be regulated at the transcriptional level by the binding of transcription factors in its promoter region (18, 47), we characterized the pig *CRP* promoter region in order to assess the level of conservation between human and cow species. A highly conserved region in *Sus Scrofa* chromosome 4 (SSC4) at position 90,782,578-90,782,833 bp was identified when compared to the human (GRCh38 1:159,714,589-159,716,089) and cow (ARS-UCD1.2 3:9,982,001-9,983,501) *CRP* promoter regions (**Supplementary Figure 1**). In addition, we performed an *in-silico* characterization of transcription factor binding sites (TFBSs) in the pig *CRP* promoter region, identifying four TFBSs conserved between pig and cow, four more between pig and human and three binding motifs maintained in all three species (**Supplementary Figure 1**).

**Figure 1:**
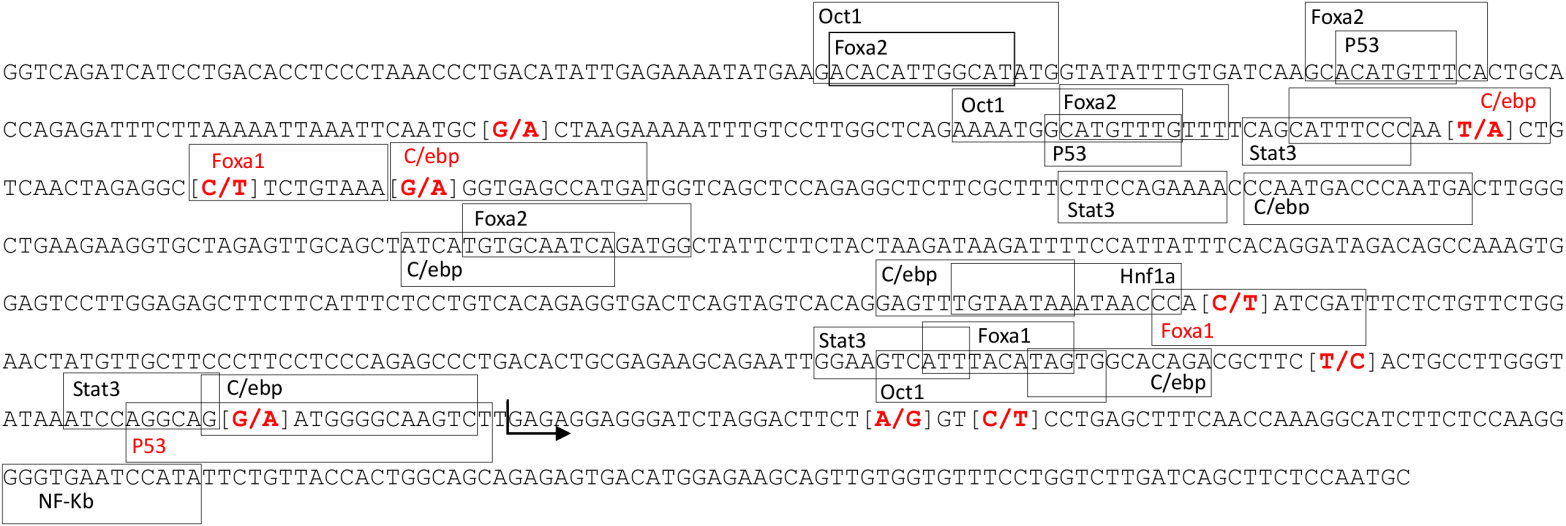
Position of the transcription factor binding sites located in the CRP promoter region. Marked in red are the SNPs found in the studied population and the transcription factors binding sites affected by the SNPs. The arrow marks the start of CRP-202 exon 1.

A total of 133 polymorphisms, most of them associated with plasma CRP levels, were identified in the *CRP* gene region in the WGS data from 100 Duroc pigs (**Supplementary Table 1**). The most significantly associated variant was rs327446000 (4:90,800,879 bp), located in the 3’ untranslated region (UTR) of *CRP*. Furthermore, we identified five polymorphisms in the pig *CRP* promoter region affecting the binding sites for C/EBP, FOXA1 and p53 transcription factors (**Figure 1**).

### 3.2 GWAS for CRP levels in serum and fine mapping revealed associated polymorphisms in the 3’UTR and upstream the promoter region of *CRP*

**Figure 2:**
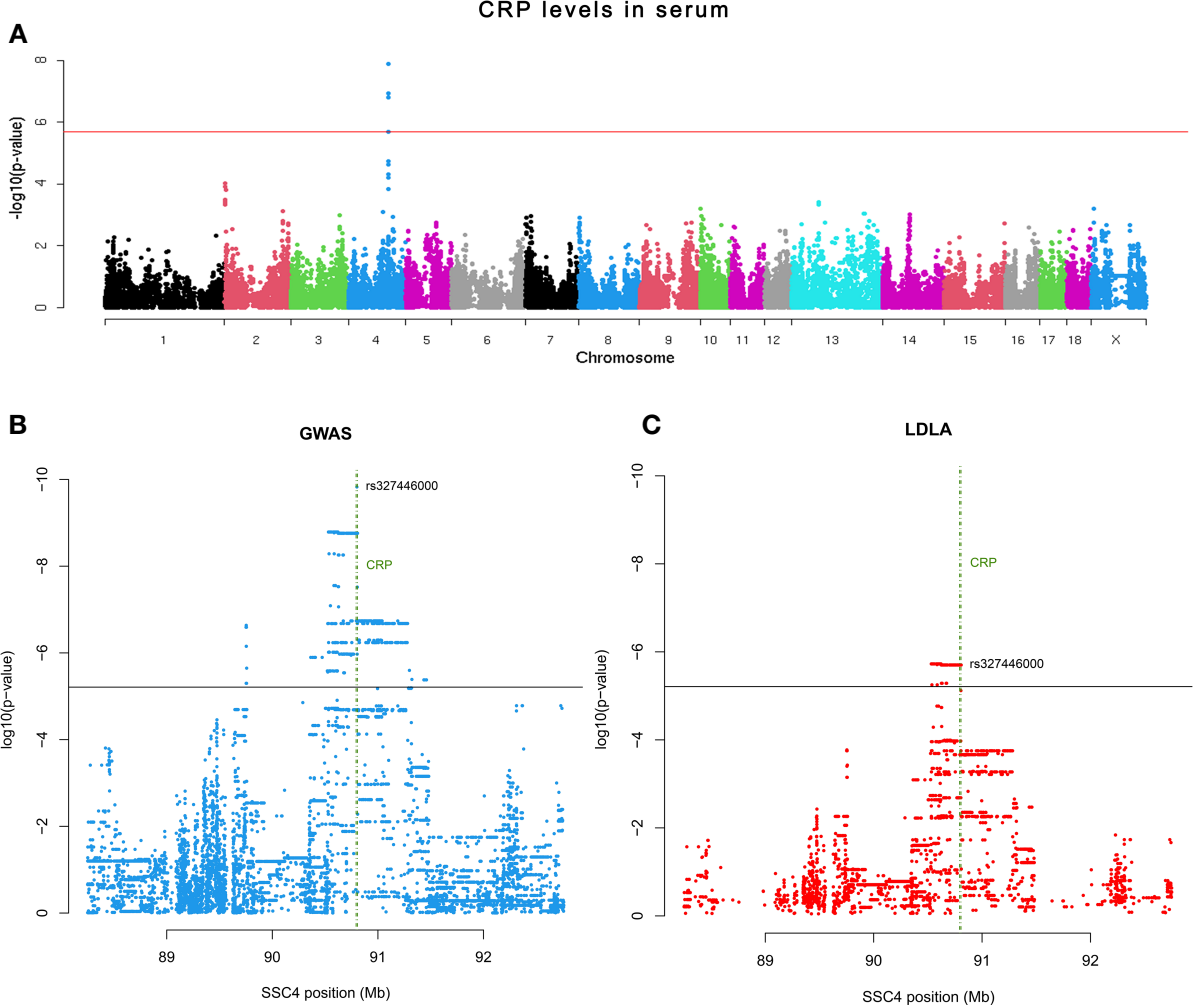
**(A)** Manhattan plot representing the association analysis between the CRP levels in serum and SNPs distributed along the pig chromosomes. **(B)** Scatter plot depicting P-value distribution of the CRP QTL at chromosome level. **(C)** Scatter plot depicting P-value distribution of the CRP QTL LDLA. Horizontal lines indicate the adjusted significance threshold (≤ 0.05). Vertical green lines encompass the CRP gene location.

The association between *CRP* polymorphisms and the variation of serum CRP levels was explored through GWAS with 42,641 SNPs plus the rs327446000 SNP from 432 Duroc pigs. A genomic region in SSC4 at 90.54-90.80 Mb was associated with serum CRP levels, with rs327446000 being the most significantly associated genetic variant (**Table 1**; **Figure 2A**).

**Table 1:** Significant polymorphisms associated to the CRP levels in serum: position, minimum allele frequency and allele substitution effect significance.

Name	Chr	Bp position	MAF	*P*-value	FDR
**rs327446000**	4	90800879	0.148	1.33 x10^-8^	0.00056832
**rs81233340**	4	90535929	0.147	1.62 x10^-7^	0.00172178
**rs81382318**	4	90598142	0.147	1.62 x10^-7^	0.00172178
**rs80958253**	4	90804626	0.217	1.16 x10^-7^	0.00172178
**rs81285109**	4	90736666	0.101	1.99 x10^-6^	0.01699241

Further exploration of the *CRP* QTL was performed by both GWAS and LDLA analyses using SSC4 genotypes from 19,610 SNPs comprising 4Mb (2Mb up and 2Mb down) of the previously declared associated genomic region. According to the GWAS results, a total of 1,482 SNPs located within a genomic region in SSC4 at 89.7-91.29 Mb were associated to the phenotype, being the top signal located at 90.80 Mb of SSC4 (rs327446000; *P*-value = 1.49 x 10^-10^) inside *CRP* (**Figure 2B**). In contrast, the LDLA study revealed a total of 483 significant signals which reduced the previous region to 90.53-90.80 Mb and positioned the maximum association at 90.53-90.62 Mb of SSC4 (*P*-value = 1.88 x 10^-6^) upstream of *CRP* (**Figure 2C**).

### 3.3 Expression GWAS for *CRP* isoforms also revealed an associated region upstream of the *CRP* gene

To identify potential functional variants affecting the expression of *CRP*, an eGWAS analysis was performed using 25,315,878 genetic variants and the expression of *CRP* isoforms in 300 pigs (n=100 Landrace, n=100 Large White, and n=100 Duroc).

**Figure 3:**
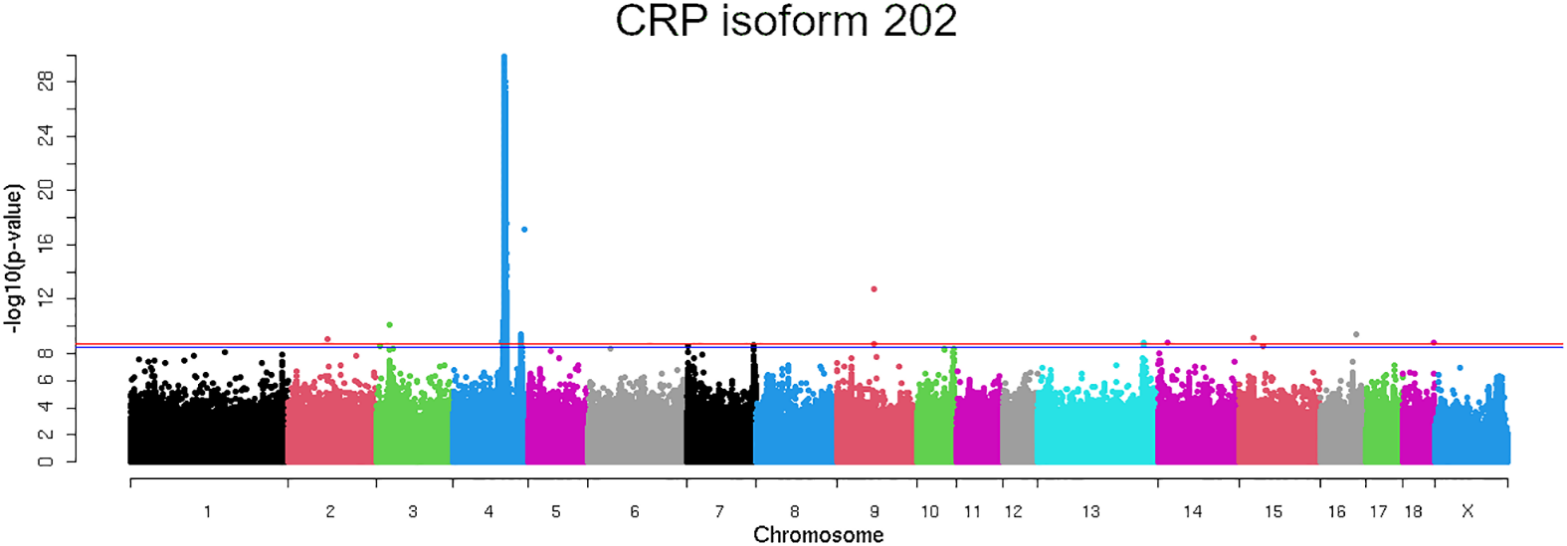
Manhattan plot representing the association analysis between the CRP mRNA expression of the isoform 202 and polymorphisms distributed along the pig chromosomes. Red line indicates the genome wide significance threshold (FDR ≤ 0.05).

A strongly associated region in SSC4 at 86-93 Mb for the expression of *CRP* isoform 202 (ENSSSCT00000054270.2) was identified (**Figure 3**), whereas not significantly associated regions in SSC4 were identified for the other *CRP* isoforms. A total of 8,250 polymorphisms were found associated (FDR ≤ 0.05) along SSC4. The top variants (adjusted *P*-value = 3.40 x 10^-23^) were rs793561911 and rs713631040, located in the positions 4:90,743,523 bp and 4:90,743,532 bp respectively, around 39.3 Kb upstream the *CRP* gene (**Table 2**; **Supplementary Table 2**).

**Table 2:** The 10 most significant polymorphisms associated to the CRP expression levels: position, alleles, minimum allele frequency and allele substitution effect significance.

Name	Chr	Bp Position	A1	A2	MAF	N°	*P*-value	FDR
**rs793561911**	4	90743523	TCTTCTGTTTGTGGGACCGGCC	T	0.2417	300	1.34 x10^-30^	3.40 x10^-23^
**rs713631040**	4	90743532	G	A	0.2417	300	1.34 x10^-30^	3.40 x10^-23^
**rs330141279**	4	90743549	G	A	0.24	299	2.24 x10^-30^	5.67 x10^-23^
**rs334016742**	4	90796210	G	C	0.245	300	2.53 x10^-30^	6.39 x10^-23^
**rs325087855**	4	90681003	C	T	0.2333	300	3.80 x10^-30^	9.63 x10^-23^
**rs328995216**	4	90744910	T	G	0.2467	300	4.14 x10^-30^	1.05 x10^-22^
**rs338992142**	4	90743570	A	G	0.2408	298	4.63 x10^-30^	1.17 x10^-22^
**rs322057211**	4	90673382	G	A	0.2391	299	6.66 x10^-30^	1.69 x10^-22^
**rs693961338**	4	90801224	C	T	0.238333	300	7.08 x10^-30^	1.79 x10^-22^
**rs331519256**	4	90679310	C	T	0.244147	299	7.12 x10^-30^	1.80 x10^-22^

### 3.4 Rs793561911 and rs713631040 polymorphisms are located in a conserved enhancer region for *CRP*

The polymorphisms most significantly associated with *CRP-202* isoform expression were located in an intergenic region conserved between several pig breeds and other species such as cow, sheep and horse (**Supplementary Figure 2**). The alignment of this region in the porcine genome (SSC4:90,743,525-90,743,526) against the human genome revealed the presence of a human conserved sequence corresponding to an enhancer element (ENSR00000931831). This distal enhancer region was also well conserved in the cow genome (ARS-UCD1.2 3:9,937,919:9,938,698). In the three species, this region was located at 39-44 Kb upstream of *CRP*. To better understand the regulatory role of this region on *CRP-202* expression, an *in-silico* characterization of TFBSs was performed. Remarkably, a total of 26 TFBSs known to regulate *CRP* expression were found within this region. Eight of them were shared with the cow genome and another site was conserved in both cow and human genomes (**Supplementary Figure 3**).

**Figure 4:**
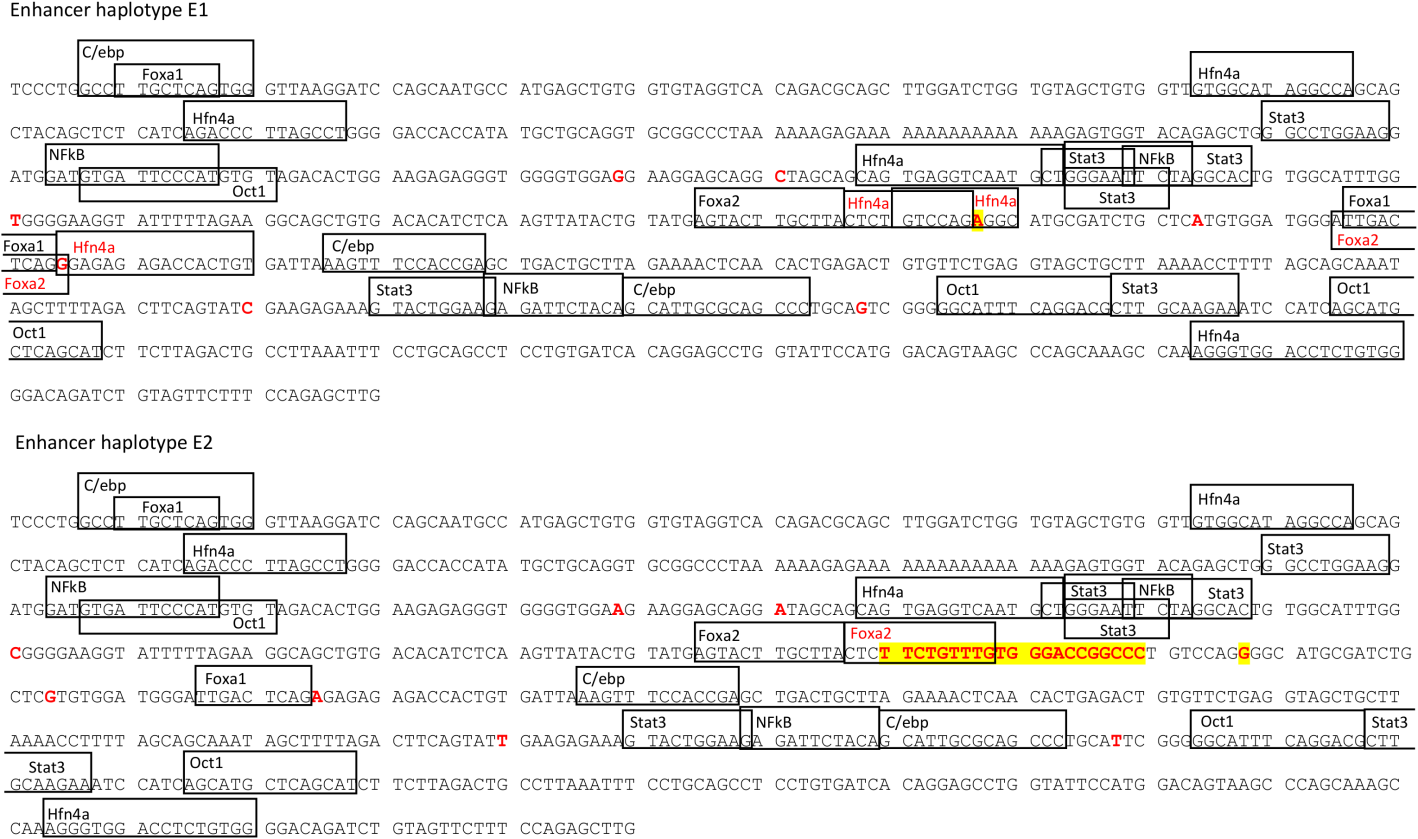
Position of the transcription factor binding sites located in the enhancer region for the different transfected sequences. Marked in red are the polymorphisms found in the studied population and the TF binding sites affected by the changes in the sequence. Highlighted in yellow are the top polymorphisms found in the eGWAS (rs793561911 and rs713631040 respectively).

In depth analysis of this region revealed that rs793561911 and rs713631040 variants were located within the binding site of the transcription factor HNF4α. Furthermore, the insertion allele of the rs793561911 polymorphism (-/TTCTGTTTGTGGGACCGGCCC) generated a binding site for the FOXA2 transcription factor. Seven other SNPs were found in this conserved region in the studied population (**Figure 4**). Moreover, a third polymorphism (rs338992142) in the position 4:90,743,570 bp was found to be in the potential binding site of both transcription factors HNF4α and FOXA2. This SNP was also found to be associated with *CRP* expression in the eGWAS (**Table 2**). These three polymorphisms and a fourth significant SNP (rs330141279) located in the position 4:90,743,549 bp were in total linkage disequilibrium resulting the following haplotype combinations: Haplotype E1: Ø – A – A – G and Haplotype E2: TTCTGTTTGTGGGACCGGCCC – G – G – A. **Figure 4** shows the enhancer regions of animals with haplotypes E1 and E2.

**Figure 5:**
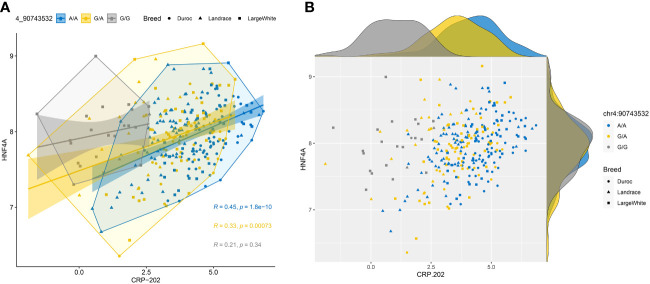
Correlation plots between HNF4α and CRP-202 mRNA expression levels by breed and rs713631040 (SSC4:90,743,532 bp) alleles. **(A)** Scatter plot with regression lines, confidence intervals, concentration polygons, and correlation coefficients. **(B)** Scatter plot with marginal density plots.

When we studied the correlation between *CRP-202* mRNA expression and *HNF4α* and *FOXA2* mRNA expression in 300 pigs, a higher correlation was observed between *HNF4α* and *CRP* gene expression when compared to *FOXA2- CRP-202* correlation (**Table 3**). Remarkably, Duroc and Landrace animals presented higher correlations between *HNF4α* and *CRP-202* mRNA levels than Large White animals (r_p_ = 0.515 for Duroc, r_p_ = 0.47 in Landrace and r_p_ = 0.297 in Large White), in accordance with their higher frequency of the A allele in rs713631040, which creates a binding site for HNF4α (**Figure 5**, **Table 3**). A similar correlation pattern between *HNF4α* and *CRP-202* was observed when all animals were classified according to the genotypes of rs713631040, with higher correlation levels observed for the AA genotype when compared to GA and GG.

**Table 3:** Correlation coefficients of CRP-202 mRNA expression with HNF4A and FOXA2 mRNA expression in liver by breed and rs713631040 (SSC4:90,743,532 bp) genotype.

Liver mRNA CRP-202	HNF4α	FOXA2	Allele A frequency (rs713631040)
**All**	0.31	0.178	
**Duroc**	0.515	0.16	0.825
**Landrace**	0.47	0.076	0.87
**Large White**	0.297	0.069	0.58
**rs713631040 A/A (n=178)**	0.455	0.16	
**rs713631040 G/A (n=99)**	0.334	0.076	
**rs713631040 G/G (n=23)**	0.21	0.069	

### 3.5 A distal enhancer upstream of the porcine *CRP* gene mediates the acute phase induction of *CRP* in HepG2 cells

**Figure 6:**
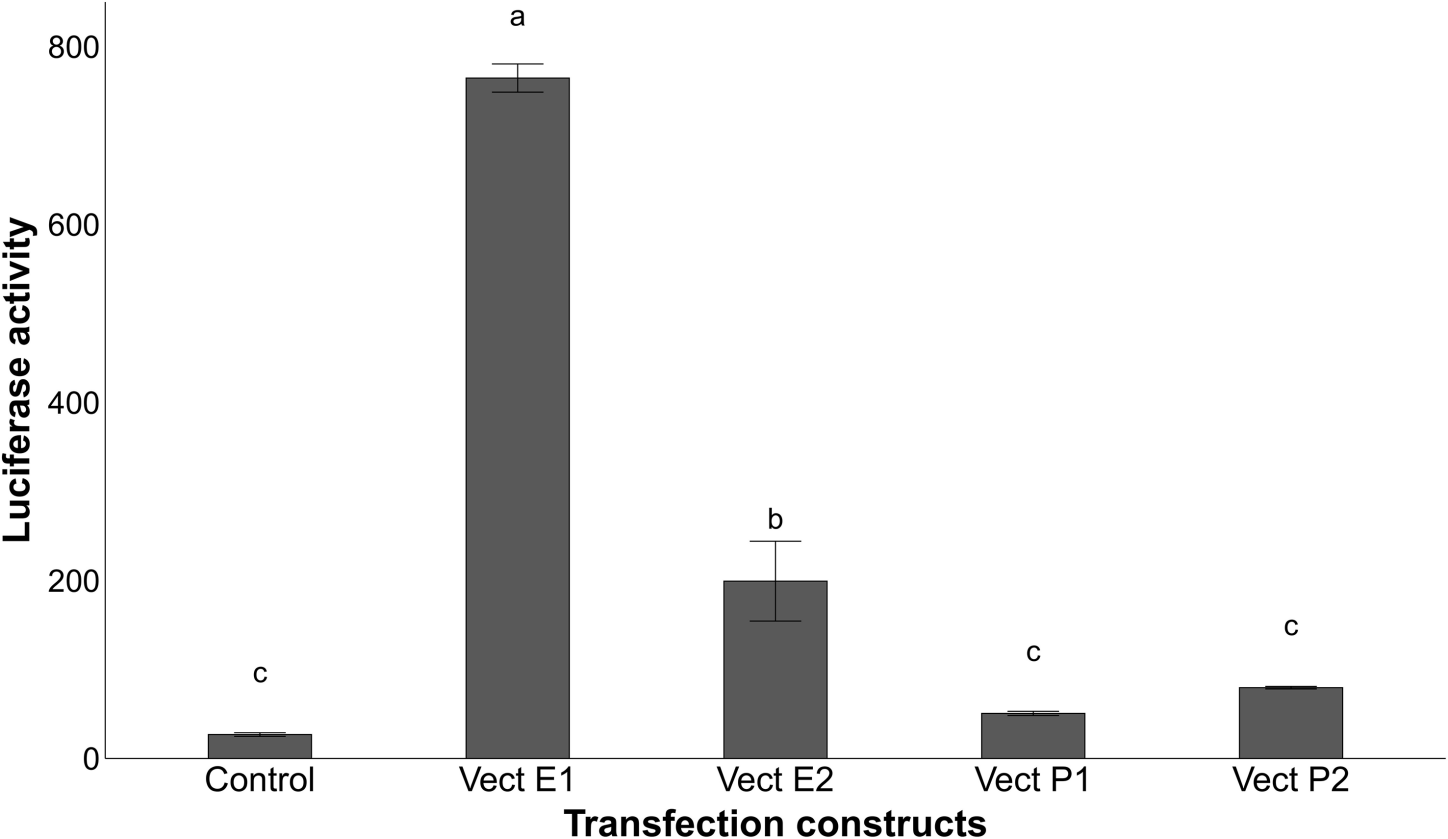
Relative luciferase activity of the transfected HepG2 cells treated with 10ng/ml IL-6 and 1ng/ml IL-1β for 24h after normalization with the cotransfected firefly luciferase activity. Vectors P1 and P2 are the constructs containing the different sequences of the promoter region alone, while vectors E1 and E2 have the respective enhancer haplotype inserted upstream of the promoter constructs P1 for the first haplotype and upstream of vector P2 for the second haplotype. Values with different superscript letters (a–c) indicate significant differences between groups (P-value ≤ 0.05), obtained by pairwise comparison by Fischer’s least significant difference (LSD) analysis adjusted with Bonferroni correction.

To examine whether the pig proximal promoter is sufficient to mediate the induction of porcine *CRP* by IL-6 and IL-1β and whether the identified polymorphisms in its core promoter region may affect acute phase induction, luciferase reporter assays were carried out. Promoter activity was measured for two vectors containing different haplotypes (P1 and P2) of pig *CRP* promoter region in transfected HepG2 cells induced with IL6 and IL1-β. No substantial increase in luciferase activity (**Figure 6**) was observed in transfected cells with both CRP promoter constructs, suggesting that the promoter alone is insufficient for the acute phase induction of porcine *CRP*.

To further understand the functional contribution of the putative enhancer region associated to the expression of *CRP*, two sequences with different haplotypes (E1 and E2) of the enhancer were inserted upstream of *CRP* proximal promoter constructs. The inclusion of the enhancer sequences in the transfected vectors increased the induction of luciferase activity in HepG2 cells (**Figure 6**), revealing the involvement of this distal regulatory element in the acute phase induction of pig *CRP* by IL-6 and IL-1β. Furthermore, the vector containing the haplotype E1 (Ø – A – A – G) generated higher levels of luciferase activity than the rest (**Figure 6**), confirming the regulatory role of rs793561911 and rs713631040 on *CRP* gene expression.

## 4 Discussion

CRP is known to be highly conserved between different species (48). Multiples studies in humans have described the regulatory molecular mechanisms controlling *CRP* gene expression in liver, as well as identified mutations associated with CRP blood levels and cardiovascular disease risk (6, 49, 50). Since pig represents an ideal model for human diseases (51, 52), in the present work we have delved into the genetic architecture and regulatory mechanisms involved in *CRP* gene expression in pigs. Furthermore, the identification of functional genetic variants associated to CRP blood levels could be valuable to improve the accuracy of genomic selection for immunocompetence in pigs.

Previous studies in humans and pigs have identified polymorphisms in the 3’UTR of *CRP* affecting serum levels of CRP (53–55). A GWAS study in our Duroc population also pointed out the polymorphism rs327446000 in the 3’UTR of *CRP* as the genetic variant most significantly associated with CRP serum levels. However, the haplotype-based approach maximized a region at 90.53-90.62 Mb in SSC4 as the most associated region with CRP serum levels. In addition, eGWAS analysis using 300 animals of different breeds identified a region at 86-93 Mb in SSC4 as the most associated with *CRP* mRNA expression levels in liver. Although we cannot discard the role of the 3’UTR region in the variation of CRP serum levels, our eGWAS and fine-mapping results pinpointed a genomic region located upstream of *CRP* gene associated with both mRNA expression and protein CRP levels.

A more detailed analysis of this region revealed the presence of a putative enhancer element conserved between human and cow species, and containing transcription factor binding sites for STAT3, C/EBP, NF-kB, HNF4α, OCT-1 FOXA1 and FOXA2. These transcription factors have been widely described as being required for the constitutive expression and/or acute phase induction of *CRP* (21, 22, 56–59). Remarkably, a recent study performed in humans identified an enhancer (E1) located 37.7 Kb upstream of the *CRP* promoter. Transcription factors STAT3, C/EBP-β, and USF1/2 appear to mediate the regulatory effects of E1 acting in conjunction with CRP proximal promoter for the acute phase induction by IL-6 and IL-1β of human *CRP* (16, 25, 26, 60). Furthermore, the constitutive expression of human CRP at the basal state seems to be mediated by promoter binding of transcription factors such as HNF-1, HNF-3 and OCT-1 (21, 56). Comparative analysis between human, cattle and porcine CRP promoter sequences also revealed a high level of sequence conservation, with transcription factor binding sites for FOXA2, HNF-1 and STAT3 preserved in the three promoter regions. Furthermore, the porcine promoter sequence shared target sites with its bovine counterpart for C/EBP, c-Rel and p53 transcription factors, and, in different locations, with the human for C/EBP, p53, OCT-1 and FOXA1. It is worth noting that the identified OCT-1 binding site conserved in pigs and humans was previously found by Voleti et al. in 2012 (22) as a modulator of *CRP* expression in humans by positional competition with other binding sites in the region.

Similar to the results previously reported by Wang et al. (26), the interaction of the pig distal enhancer element with the *CRP* proximal promoter was required for the acute phase induction of porcine *CRP* by IL-6 and IL-1β, suggesting an evolutionary conservation of regulatory mechanisms involved in *CRP* expression between pigs and humans. These results are in agreement with the similar functions of this protein in humans and pigs compared to mouse CRP (48).

Several polymorphisms located in putative binding-regions of transcription factors and associated to *CRP* mRNA expression and protein levels were identified in the proximal promoter and distal enhancer of porcine *CRP*. In the proximal promoter region, five out of 17 described genetic variants were disrupting putative binding-sites for C/EBP, FOXA1 and p53. C/EBP has been described as an important transcription factor activated by IL-6 and necessary for the induction of *CRP* expression (60, 61). However, we did not observe differences in luciferase activity in transfected HepG2 cells with vectors containing different promoter haplotypes after cytokine stimulation, suggesting that the allelic variation in these putative C/EBP binding sites did not have a substantial effect in the expression of porcine *CRP*. By contrast, among the seven polymorphisms found in the porcine enhancer region, rs793561911, rs713631040 and rs338992142 were located within putative binding sites for HNF4α and FOXA2, and potentially disrupting their regulatory effect. In fact, our luciferase assay showed a significant increase in luciferase activity in HEPG2 cells transfected with the enhancer haplotype that conserved the HNF4α binding sites (E1), which is in accordance with the correlation observed between *CRP* and *HNF4α* mRNA expression levels in liver.

Hepatocyte nuclear factor 4 alpha (HNF4α) encodes for a protein that controls the expression of several hepatic genes, HNF1α among them, and plays a role in liver development (62, 63). Sucajtys−Szulc et al. (19) revealed a coordinated upregulation of both hepatic nuclear factors, as well as IL-6 and CRP in livers of rats affected with either chronic renal failure or lipopolysaccharide-induced inflammation.

In the light of the above, our results describe for the first time the role of a distal enhancer in the acute phase expression of porcine *CRP*. Our analysis on CRP serum levels was limited to a closed commercial Duroc line, which is reflected in a high linkage disequilibrium. A larger sample size including other pig breeds and commercial lines would reduce the presence of large associated blocks and allow the identification of the causal implicated variants. Further functional analyses are warranted to better understand the regulatory mechanisms involved in *CRP* expression as well as to locate the causal mutation(s).

Finally, taking into account the strong similarities between porcine and human *CRP* regulation, this work improves the understanding of the complex mechanisms governing *CRP* expression in both species and reiterates the advantages of using the pig as a biomedical model for inflammation and cardiovascular diseases in humans. In addition, the identified functional polymorphisms can be used in pig breeding programs to improve the immunocompetence profile of the herd.

## Data availability statement

The datasets presented in this study can be found in online repositories. The names of the repository/repositories and accession number(s) can be found below: https://data.faang.org/, PRJEB58030 and PRJEB58031.

## Ethics statement

The animal study was approved by Institut de Recerca i Tecnologia Agroalimentàries (IRTA) Ethical Committee. The study was conducted in accordance with the local legislation and institutional requirements.

## Author contributions

MB designed the study. MB, M-JM and MCAMB supervised the generation of the material animal. MB, OG-R, YR-C and RQ performed the sampling. MB, OG-R, TJ-J and CH-B carried out the laboratory analyses. TJ-J, CH-B, DC-P, AE-C, MB and RQ analysed the data. CH-B and MB interpreted the results and wrote the manuscript. All authors contributed to the article and approved the submitted version. 
