# Supplementary material for: Mutations on a conserved distal enhancer in the porcine C-reactive protein gene impair its expression in liver
Source: Front Immunol. 2023 Sep 14;14:1250942. doi: 10.3389/fimmu.2023.1250942 (PMC10539928; doi:10.3389/fimmu.2023.1250942)
Supplement: Supplementary file 1 [file DataSheet_1.docx]

Supplementary Material

Mutations on a conserved distal enhancer in the porcine C-reactive protein gene impair its expression in liver.

Carles Hernández-Banqué^1^, Teodor Jové-Juncà^1^, Daniel Crespo-Piazuelo^1^, Olga González-Rodríguez^1^, Yuliaxis Ramayo-Caldas^1^, Anna Esteve-Codina^2,3^, Marie-José Mercat^4^, Marco C.A.M. Bink^5^, Raquel Quintanilla^1^, Maria Ballester^1^*

*** Correspondence:**
Maria Ballester:
[maria.ballester@irta.cat](mailto:maria.ballester@irta.cat)

Supplementary table 1. Position of the polymorphisms found in the CRP gene and its regulatory region and level of association with CRP serum concentration.

Supplementary table 2. Position of the 50 most significant SNVs (over the genome wide significance threshold) associated to the CRP expression in the eGWAS study.

Supplementary Figure 1. Alignment of the characterized porcine promoter region and the reference human and bovine CRP promoters, and position of the conserved transcription factor binding sites.

1 130

cow GGAGGCAG AATATGTAAG TAAAGATTCA GAAAGTTGGA GATGGGAGGA TGTGTAAAGA TGTCAGTTTG GATTTTATAG CAGGAAGACC ACATTAATAG TTTTGATTTA ACAAAGGACA GTATGTCTCC

pig AAGGAGGCAG AATATGTGAG TAAAG-TGCA GAAAGATTGG TACACTTGGA -GTGGGAGGA TGT--GTAAG GTTAT----- CAGCTAGCC- ---------- ------TTTA TAATAGGAAG AT--------

human

Consensus ..ggaggcag aatatgt.ag taaag.t.ca gaaag.t.g. .a.....gga .gtg..a.ga tgt..gt..g g.t.t..... cag..ag.c. .......... ......ttta ..a.agga.. .t........

131 260

cow TTCAAGGAAA TGTTGGGGTA GTTAGTCTAG AGCTGATGTG GTGGTTCCAT AGTCAATTAT CCAGGTTCCA TCTATCTGCA ACCTCAAATG TGCATCTCAC CCATGGTGGT CTAAAAGTGA --TACTCTAG

pig --CAAATTAA TGTTCGAGTA GGTGGTCTAG AGCCGTTATA GCGGTTCCAG AACCAATTAT CCAGGCTCCA TCTATCTGCC AGCTTCAGCG TGCATCTTAC CCACGGTGGT C-AAAGGTGA --TATTCTAG

human CAGG CTCCATCTAT CTTGTTGCCA T------GCC AACCTCAACA TGCAGCTTAC ACTTGCTGGC CTGAAAGTGA ACTATTCCAT

Consensus ..caa...aa tgtt.g.gta g.t.gtctag agc.g.t.t. g.ggttCcag a.cCAatTAT CcaGgttCCA TctatctGCc A.CttcAacg TGCAtCTtAC cCatGgTGGt CtaAAaGTGA ..TAtTCtAg

P53

261 390

cow CTCCTATTAT CATGTCTGAA TACTAGACAG ATGGCAGGAG GGG-AAGTGG AAAGGAAGAG CACAAGAGAA GTCTTTAAAG GGCGCAACCT GGAAAATGTC CTTATTCCTT CTGTTCATAT CTCATTGGCC

pig CTCCTATCAT CATGCCT-CA CACCAGGCAA AGGGAAGG-- GAG-AAGTGG AA-GGTAGAG CAGATGCCTT CACTTTAAAG GGCACAACCT GGAAATCATC CCTCTTACTT CTGTTCATAT CTCACTGGCC

human CTCCTGCTAT CATGACTGAA TACTAGTCAA AGGGAAAAAG GGGGAAGTGG GAGGGGAAAG CACATACCTT -TCTT-AAAG AGCACAACCT AGAGATGGTC CCTATT---- CTGTTCATAT CTCGTTGGCC

Consensus CTCCTattAT CATG.CTgaA tACtAG.CAa AgGGaAggag GgG.AAGTGG aA.GG.AgAG CAcAtgcctt .tCTTtAAAG gGCaCAACCT gGAaAt.gTC CcTaTT.ctt CTGTTCATAT CTCatTGGCC

c/EBP

391 520

cow AGAATGTAAT CACATTCTCA CTCCTAACTG CAGAGGAGGC TGTGAAGTTT AGTATTTGAC TGGGTGACTG TGTGCTCAAC CAATAGTGCT GTTCTTACGG GATGAGAAGT GGAGAATGGG CATTGTGCAG

pig AGAACATACT CACATTCTCA TTCCCAGCTG CAAGGGAGGC TGGGAAATTC A----TTGGC TAGGTGACCA TGTGCCCAAC CACCAATGCC ACTCTTACTG TAGGAGAAGT GGAGAATGGA CACTGGGGAG

human TTAATTTAAT CACATCATCA CTGCAAGCTA CAGTGGGGGC TGGGAAATTT GGTGTTTGAG GGGATGACCA AGCGCCCAAC TGAAAATGTT ATTCTTCTCT TATGAGAAGT AGAGAATGGA TGTTAGGGAA

Consensus agAAt.TAaT CACATtcTCA cTcC.AgCTg CAg.GGaGGC TGgGAAaTTt agt.tTTGac tgGgTGACca tGtGCcCAAC caa.AaTGct atTCTTac.g tAtGAGAAGT gGAGAATGGa catTggGgAg

521 650

cow CTGCCAGAAG CCTCTGCTAC AAGAGATACA GTGGCTTTTT CCTGTTTGCT TCTAATTTCT CAATAAAGAG CT-GTGATT- -GCTGTGTAT AAGAAT---- ---------- ---------- --------GG

pig CT---AGAAG CCCCTGCTAC AAAAGGTATG GTCATTCTTC TCTGATGACT TCTA-TTTCT CAATAAAGAA AAAGTGAAT- -GCTGGGAGT TCCCATC--- ---------- --------GT GGCTCAGTGG

human TGGCTAGAAG ACTCTACTAC AAAGGATACG GTGGTTCTTT TCTGATTGCT TCTAACTTCT CAATGAAAGG TGAGTTCATC AGCTGAATGA AAGAATAAAT GAGATTTTGG GGACTTGAGG AGCAAGGAGA

Consensus ctgc.AGAAG cCtCTgCTAC AAaaGaTAcg GTggtTcTTt tCTGaTtgCT TCTAatTTCT CAATaAAgag ..aGTgaaT. .GCTG.gtgt aagaAT.... .......... ........g. .gc...g.Gg

651 780

cow ATGAGATTCT GG-GGGTTTG AG-GAGACAG GA------GG AGC--TAAAT AGCCA--TT- ----TGAAGA CTGGATTAAA TA-----GGA ATCTATAGTA -AGATTGGCT ------TCCC AGC-CAATGC

pig AAACAAATCT GACTAGTATC TGTGAGGATG TA------GG TTCAATCCCT GGCCT--CAC TCAGTGGGTT AAGGATCCAG TGTTTCTGTG AGCTGTGGTG TAGGTTGCAG ------GTGC AGCTCAGATC

human AGGAGTATAA TAGTCATTTT GAAGAGTGAG TTAAGTAGGG AACTGTAGTA AGATTGACAG ACAGTGTGGA GGGATTACTT GAATCTTGTG AATAGAGGAA AGAGTAGAAT CAGATTATCC TGACTCCTGC

Consensus A.gagaaTct ga.t.gTtT. .g.GAG..aG ta......GG a.C..Ta..t aGcct..ca. .cagTG.gga ..GgaT.ca. ta.t..tGtg A.ctgtgGta .aggTtG.at .......tcC aGc.ca.tgC

781 910

cow AAGAGATACA AGGGATGTGG GTTTGATCTC TGGGT-CAG- ---GAA--GA TCCC----CT GGAGA----- -AGGAAATAG CAATCCA--- -CTCCAGTAT ---------- ---TCTTGC- --CTGGAAAA

pig CCGATTTGCT GTGGCTGTGG CTGTGGCCAG TGGCTGCAGC TCTGATTTGG TCCCTAGCCT GGGAACCTCC GAGGGTGTGG CCCTCAAAAG ACAAAAAAAT AAAATAAAAA AAATTTTGAA TGCTGAGTAT

human CTGAAGCTTT ACATATTCAG AGAAAAATGT TGGAAGAAAC TTTGATATAA TGCTATGTCT GTGATCAGGC ACACATTTTA CTGGACTTTT ACTGTCAGGG CCGTCATTTA GTGCCAAGAT GTCTAGAGAG

Consensus c.GA..t.ct a.ggaTgtgG .t.tga.c.. TGG.tgcAgc t.tGAt.tga TcCc..g.CT Gggaac...c .aggat.T.g C..tcca... aCt..aa.at .....a...a ...tcttGa. ..CTgga.A.

911 1040

cow TCCCATGGAC -AG-----A- ---G--GAGT CCATAGAGAT TTTCTATGGA CTATGCATC- CATAGGA--- ----CTGTGG GATGCAGTCT AC--AGA--- ------GTCA CAAAGAG--- --TCAGACA-

pig AAGAATGGAT GAG-----AT TTGG--GGGT AAGAAGAGAG GAGGAGAAAA ATAATCATC- CTGAAGGGTG ATTTCAGTAG GGAACGGTAC TA--AGATTG CCAGGCATCA TGGAGAA--- --ACTACTT-

human TTCTTAATAA GTGTACTCAA TTGGCTGAGA AAATGTGTCC ATGCAAAAAA CCAAACACCG CGTGTTCTCA CTCATAGATG GGAATTGAAC AATGAGAATA CTTGGACACA GGAAGGGGAA CATCACACTC

Consensus t.c.atggA. gaG.....A. ttgG..GaGt aaatagaga. .tgcaaaaaA ctAa.CAtC. C.ta.g.... .t..caGt.G Ggaac.Gtac aa..AGA.t. c..gg..tCA .gaAGag... ..tCa.act.

p53

1041 1170

cow ----GAA--- -----CTGAA GGA-----CT GGG-CACATC ACCTTGACTC TTCCCTGAAC CTTGACAT-- ---------- -GAAG-AAAC TC---TGACA TATGGTAAGT TTGTGATCAG TTACATGTTT

pig ----GAATTT TGTGAATAAA GGAAATGGCA GGGTCAGATC ATCCTGACAC CTCCCTAAAC CCTGACATAT TGAGAAAATA TGAAG-ACAC AT---TGGCA TATGGTATAT TTGTGATCAA GCACATGTTT

human TGGGGACTGT TGTGGGGTGG GGGGAGGGGG GAGGGATAGC ATTAGAAGAT ATACCTAATG CTAAATGATG AGTTAATGGG TGCAGCACAC CAGCATGGCA CATG-TATAC ATATG-TAAC TAACCTGCA-

Consensus ....GAat.t tgtg..t.aa GGa.a.ggc. GgG.cA.AtC Atc.tgAcac .TcCCTaAac CttgAcat.. .g..aa.... tGaAG.AcAC .....TGgCA tATGgTAtat tTgTGaTcA. t.ACaTGttt

FOXA2

1171 1300

cow CACTGGACCA GAGATTTCTT TGCAATGAAA CTCAATTGGC TAAGAAAAAA TTGTCCTTGG CTCTGTGGAT GACATGTTTG TTTTTGGCAT TTCCCAATTT GTGAACCAGA GGCATCTGAA AAGGGTCGGC

pig CACTGCACCA GAGATTTCTT AAAAATTAAA TTCAATGCGC TAAGAAAAAT TTGTCCTTGG CTCAGAAAAT GGCATGTTTG TTTTCAGCAT TTCCCAATCT GTCAACTAGA GGCCTCTGTA AAGGGTGAGC

human CATTGTGCAC ATGTACCCTA AAACTTAAAG TATAATAATA ATAAAAAAAT GTGTCCATGG CTCTGGGAGG AGCATGTTTG TTTTCCTCAT TTCCCAGTCT GTAAATAAGC A--AATTGAA AGGGGTTAGT

p53

Consensus CAcTG.aCca gaGatttCTt aaaaaT.AAa ttcAAT..gc taAgAAAAAt tTGTCCtTGG CTCtG.gaat ggCATGTTTG TTTTc.gCAT TTCCCAaTcT GT.AAc.AGa ggcatcTGaA AaGGGT.aGc

1301 1430

cow AATGATGTTC ATCTCCAGAA GCTATCCTC- -----CATGA AACACCAA-- -------GAC TTGGGCTGAA GTGGGTGCTA GAGATATA-C TATGATATGT TCTCAGATGG CCATG-TTCT ATTAAGATT-

pig CATGATGGTC AGCTCCAGAG GCTCTTCGCT TTCTTCCAGA AAACCCAATG ACCCAATGAC TTGGGCTGAA GAAGGTGCTA GAGTTGCAGC TATCATGTGC AATCAGATGG CTATTCTTCT ACTAAGATAA

human GATAATGTCC ATCTCCAGAA GCTGTCAGAT TTCCTTTGTC AAACTCTAT- -------GAT TTGGGCTGAA GTAGGTGTTG GAGAGGCAGC TACCACGTGC ACCCAGATGG CCACTCGTTT AATATGTTAC

Consensus .ATgATGttC AtCTCCAGAa GCT.Tccgct ttc.tc..ga AAaccCaAt. .......GAc TTGGGCTGAA GtaGGTGcTa GAGatgcAgC TAtcAtgTGc actCAGATGG CcAttctTcT A.TAaGaTa.

HNF1A

1431 1560

cow --TTTAAATT A-TTTTCTCA AGATAAATA- CCCAAGTGGA ACTCTCAGGG AGCTTCTTCA TTTTTCCTGG CATAAAGAAT TGGTGATCCC ATAGTCACAG GAGCTTGTAA TAAATAACCA ACATTGATTT

pig GATTTTCCAT T-ATTTCACA GGATAGACAG CCAAAGTGGA GTCCTTGGAG AGCTTCTTCA TTTCTCCTGT CACAGAG--- --GTGACTCA GTAGTCACAG GAGTTTGTAA TAAATAACCC ACATCGATTT

human CATTTCCCAT TATTTTCGCA GGATAGATAG CCAAAGTGGA GCCCTGAGAG A-TTTCTTCA TTTTTCCTGT CATAAAGAAT TGGTAATTCA GTAGTCATAG GAGTTTGTAA TAAATAACTC ACATTGATTT

c/EBP

Consensus .aTTT.ccaT t.tTTTC.CA gGATAgAtAg CCaAAGTGGA gccCT.aGaG AgcTTCTTCA TTTtTCCTGt CAtAaAGaat tgGTgAttCa gTAGTCAcAG GAGtTTGTAA TAAATAACcc ACATtGATTT

1561 1690

cow CTCTGTTCCA GAATTATTTT GCTTCCCTTC TTTCCAGAGC CCTGACGCTT GCTGAGAAAG GTAGTATTGG AAAATCATTT ACGT-GTGGC ACAGACTCCC CCACTGCCTT GGATATAAAT CCAGGCAGGA

pig CTCTGTTCTG GAACTATGTT GCTTCCCTTC CTCCCAGAGC CCTGACACT- ---GCGAGAA GCAGAATTGG AA-GTCATTT ACATAGTGGC ACAGACGCTT CTACTGCCTT GGGTATAAAT CCAGGCAGGA

human CTCTGTTCTG AAATAATTTT GCTTCCCCTC TTCCCGAAGC TCTGACACCT GCCCCAACAA GCAATGTTGG AAAATTATTT ACATAGTGGC GCAAACTCCC TTACTGCTTT GGATATAAAT CCAGGCAGGA

Oct 1

Stat3

Consensus CTCTGTTCtg gAAttATtTT GCTTCCCtTC tTcCCagAGC cCTGACaCtt gc.gcgA.Aa GcAgtaTTGG AAaaTcATTT ACaTaGTGGC aCAgACtCcc ctACTGCcTT GGaTATAAAT CCAGGCAGGA

FOXA1

1691 1820

cow TGGAGCAGAT CTTGAGGCAA AGAATCTAGG ACTTCTGGTC GCTGAGCTTT CAGCCACAGA CATCT---CC AGAAGGGTGA ATCCAGATTT TAGAAGCATT GGTGGCAGAG ACTGACCATG GAGAGGCTGT

pig TGGGGCAAGT CTTGAGAGGA GGGATCTAGG ACTTCTAGTC CCTGAGCTTT CAACCAAAGG CATCTTCTCC AAGGGGGTGA ATCCATATTC TGTTACCACT GGCAGCAGAG AGTGAC-ATG GAGAAGCAGT

human GGAGGTAGCT CTA-AGGCAA GAGATCTAGG ACTTCTAGCC CCTGAACTTT CAGCCGAATA CATCTTTTCC AAAGGAGTGA ATTCAGGCCC TTGTATCACT GGCAGCAGGA CGTGACCATG GAGAAGCTGT

Consensus tGggGcAg.T CTtgAGgcaA gggATCTAGG ACTTCTaGtC cCTGAgCTTT CAgCCaaAga CATCTt.tCC AaagGgGTGA ATcCAgattc T.gtA.CAcT GGcaGCAGag agTGACcATG GAGAaGCtGT

c-Rel

1821 1880

cow TGTGGTGTTT CCTGGTCTTG ATCAGCTTCT CTAGTGTTTC AGGCCAGACA GGTAGGGGCT

pig TGTGGTGTTT CCTGGTCTTG ATCAGCTTCT CCAATGCTTT TGGCCAGTCA GGTAGGGGCT

human TGTG TTT CTTGGTCTTG ACCAGCCTCT CTCATGCTTT TGGCCAGACA GGTAAGGGCC

Consensus TGTGgtgTTT CcTGGTCTTG AtCAGCtTCT CtaaTGcTTt tGGCCAGaCA GGTAgGGGCt

Supplementary Figure 2. A) Alignment of the highly conserved intergenic region between the reference pig genome with several pig breeds and cow, sheep and horse reference genomes. B) Dendrogram of level of conservation of the intergenic region between the aforementioned species and breeds.


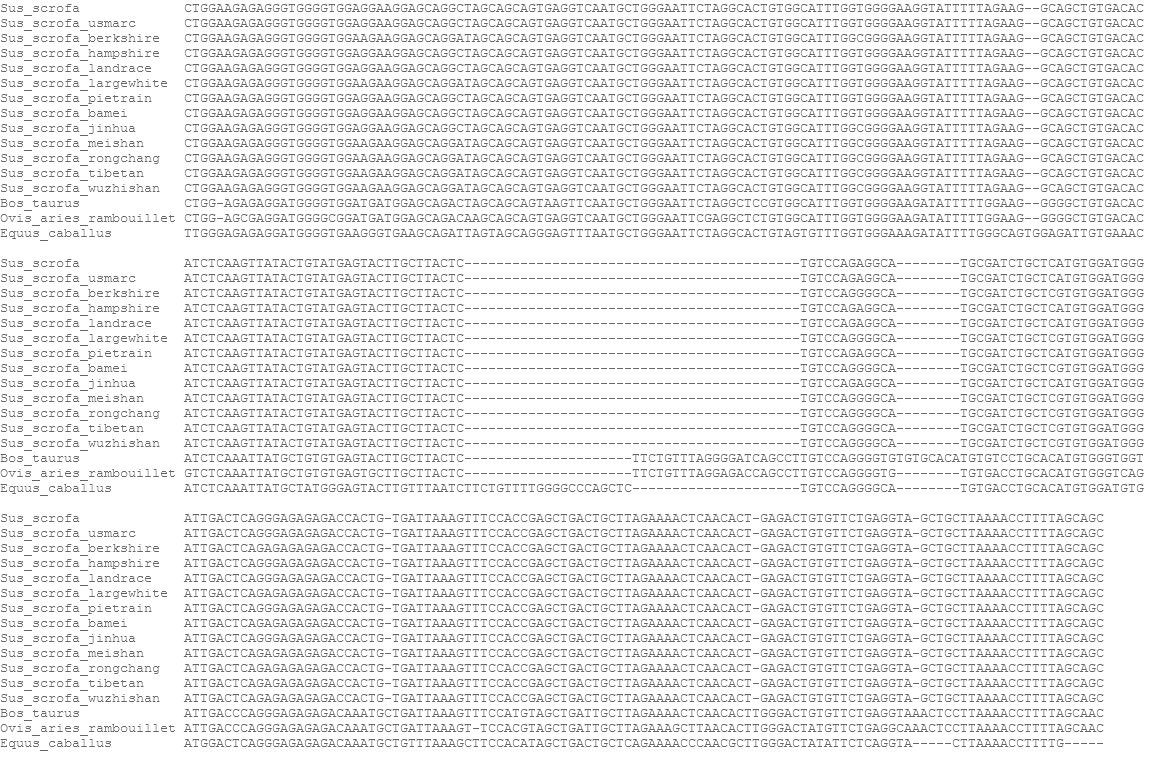


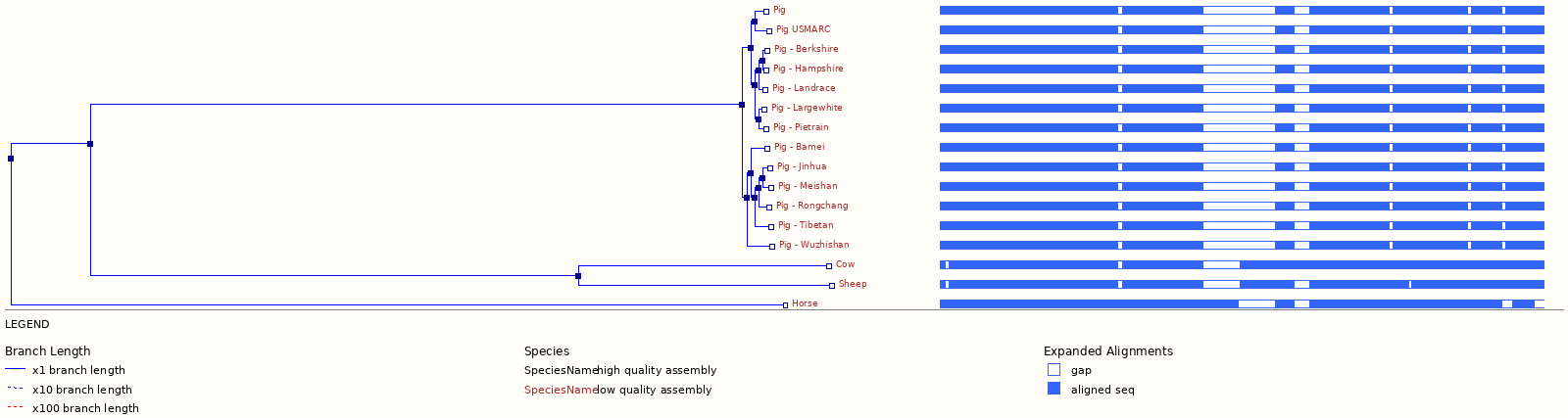


Supplementary Figure 3. Alignment of the highly conserved intergenic region between the reference pig, human and cow genomes and position of the conserved transcription factor binding sites

NF-kB p65

Stat3

Stat3

Oct 1

1 60 130

Cow GATGGGATTC ACATGTGCAG ACACTGGA-G AGAGGATGGG GTGGATGATG GAGCAGACTA GCAGCAGTAA GTTCAATGCT GGGAATTCTA GGCTCCGTGG CATTTGGTGG GGAAGATATT TTTGGAAGGG

Pig GATGTGATTC CCATGTGTAG ACACTGGAAG AGAGGGTGGG GTGGAGGAAG GAGCAGGCTA GCAGCAGTGA GGTCAATGCT GGGAATTCTA GGCACTGTGG CATTTGGTGG GGAAGGTATT TTTAGAAGGC

Human AGGAG AGATGATGGG GTGAA-GCAA GAGAAGATTA GCTGCCGTGA GTTGAATACT GGGAATTTTA GGCCCTGTGG CATTTGGTGG GGATGATATT TTGAGCAGGG

Consensus gatg.gattc .catgtg.ag acactgGaaG AGAgGaTGGG GTGgA.Gaag GAGcAGacTA GCaGCaGTgA GtTcAATgCT GGGAATTcTA GGC.CtGTGG CATTTGGTGG GGAaGaTATT TTtaGaAGGg

Foxa1/2

Foxa2

131 190 260

Cow GGCT-GTGAC ACATCTCAAA TTATGCTGTG TGAGTACTTG CTTACTCTTC TGTTTAGGGG ATCAGCCTTG TCCAGGGGTG TGTGCACATG TGTCCTGCAC ATGTGGGTGG TAT---TGAC CCAGGGAGAG

Pig AGCT-GTGAC ACATCTCAAG TTATACTGTA TGAGTACTTG CTTACTCTTC TGTTTGTGGG ACCGGCCCTG TCCAGGGG-- ------CATG CGATCTGCTC GTGTGGATGG GAT---TGAC TCAGGGAGAG

Human GGATTGTGCC ACATCTCAAA TTACACCAGG CAAGTAGTTG CTTACTATTC TGTTTATGGG ATCAACGCTG CCAAGGG--- ------CATG CTACCTGCAA ATGTGGATGG GAAGGATGAG AGAGAGAGAG

Consensus gGcT.GTGaC ACATCTCAAa TTAtaCtgtg tgAGTAcTTG CTTACTcTTC TGTTTatGGG AtCagCccTG tCcAGGGg.. ......CATG cgacCTGCac aTGTGGaTGG gAt...TGAc .cAGgGAGAG

HNF-4a

261 320 390

Cow AGACA----- ---------- ---------- ----AATGCT GATTAAAGTT TCCATGTAGC TGATTGCTTA GAAAACTCAA CACTTGGGAC TGTGTTCTGA GGTAAACTCC TTAAAACCTT TTAGCAACAA

Pig AGACC----- ---------- ---------- ----ACTG-T GATTAAAGTT TCCACCGAGC TGACTGCTTA GAAAACTCAA CACT-GAGAC TGTGTTCTGA GGTAG-CTGC TTAAAACCTT TTAGCAGCAA

Human AGAGAGAGAG ATAGAGAGAG AGAGAGAGAG AAAGAATGCT AACTGAAGCT TCTACATAGC TGACTGCTCA GAAAACCCAA CACATGGAGC TATGTTCTGA AGTAA-TTGC CTAAAACCTT T-ATCAACAA

Consensus AGAca..... .......... .......... ....AaTGcT gAtTaAAGtT TCcAc.tAGC TGAcTGCTtA GAAAACtCAA CACttGggaC TgTGTTCTGA gGTAa.cTgC tTAAAACCTT TtAgCAaCAA

Stat3

NF-kB

391 450

Cow ATTGCTTTTA GACTTCAGAA TCGCAGAGAA AATACTGGAA GAGATTCTAC AGCACTGCTC

Pig ATAGCTTTTA GACTTCAGTA TCGAAGAGAA AGTACTGGAA GAGATTCTAC AGCATTGCGC

Human ATAGTTTTTA GACTTTGGTA TGGAGGAG

Consensus ATaGcTTTTA GACTTcaGtA TcGaaGAGaa a.tactggaa gagattctac agca.tgc.c
